# Characterization of triatomine bloodmeal sources using direct Sanger sequencing and amplicon deep sequencing methods

**DOI:** 10.1038/s41598-022-14208-8

**Published:** 2022-06-17

**Authors:** Sujata Balasubramanian, Rachel Curtis-Robles, Bhagath Chirra, Lisa D. Auckland, Alan Mai, Virgilio Bocanegra-Garcia, Patti Clark, Wilhelmina Clark, Mark Cottingham, Geraldine Fleurie, Charles D. Johnson, Richard P. Metz, Shichen Wang, Nicholas J. Hathaway, Jeffrey A. Bailey, Gabriel L. Hamer, Sarah A. Hamer

**Affiliations:** 1 Department of Veterinary Integrative Biosciences, MS 4458, Texas A&M University, College Station, TX 77843 USA; 2 Department of Epidemiology and Biostatistics, Texas A&M Health Science Center, College Station, TX 77843 USA; 3 Centro de Biotecnología Genómica, Instituto Politécnico Nacional, Reynosa, TAMPS Mexico; 4 Austin Zoo, Austin, TX 78736 USA; 5 SNBL USA, Ltd., Alice, TX 78332 USA; 6 Genomics and Bioinformatics Services, Texas A&M Agrilife Research, College Station, TX 77843 USA; 7 Department of Medicine, University of Massachusetts Medical School, Worcester, MA USA; 8 Department of Pathology and Laboratory Medicine, Brown University, Providence, RI 02912 USA; 9 Department of Entomology, MS2475, Texas A&M University, College Station, TX 77843 USA; 10 Present Address: Department of Microbial Pathogenesis & Immunology, Texas A&M University Health Science Center, College Station, TX 77843 USA; 11 Present Address: Florida Department of Health, Public Health Research Unit, Tallahassee, FL 32399 USA; 12 Present Address: Charles River Laboratories, Reno, NV 89511 USA

**Keywords:** Molecular ecology, Entomology

## Abstract

Knowledge of host associations of blood-feeding vectors may afford insights into managing disease systems and protecting public health. However, the ability of methods to distinguish bloodmeal sources varies widely. We used two methods—Sanger sequencing and amplicon deep sequencing—to target a 228 bp region of the vertebrate Cytochrome b gene and determine hosts fed upon by triatomines (n = 115) collected primarily in Texas, USA. Direct Sanger sequencing of PCR amplicons was successful for 36 samples (31%). Sanger sequencing revealed 15 distinct host species, which included humans, domestic animals (*Canis lupus familiaris*, *Ovis aries*, *Gallus gallus*, *Bos taurus*, *Felis catus*, and *Capra hircus*), wildlife (*Rattus rattus*, *Incilius nebulifer*, *Sciurus carolinensis*, *Sciurus niger*, and *Odocoileus virginianus*), and captive animals (*Panthera tigris*, *Colobus* spp., and *Chelonoidis carbonaria*). Samples sequenced by the Sanger method were also subjected to Illumina MiSeq amplicon deep sequencing. The amplicon deep sequencing results (average of 302,080 usable reads per sample) replicated the host community revealed using Sanger sequencing, and detected additional hosts in five triatomines (13.9%), including two additional blood sources (*Procyon lotor* and *Bassariscus astutus*). Up to four bloodmeal sources were detected in a single triatomine (*I. nebulifer*, *Homo sapiens*, *C. lupus familiaris*, and *S. carolinensis*). Enhanced understanding of vector-host-parasite networks may allow for integrated vector management programs focusing on highly-utilized and highly-infected host species.

## Introduction

Determining bloodmeal sources of arthropod vectors can reveal vector-host interactions critical to understanding networks of pathogen transmission and guiding vector control and disease prevention campaigns. For example, mosquito host feeding patterns vary temporally, which correspond to changes in vertebrate availability and potentially the transmission of mosquito-borne viruses^1,2^. Previous triatomine research has revealed bloodmeal host utilization differences by season and instar stage; for example, in a study in Argentina, *Triatoma infestans* fed on a larger number of different hosts and took mixed meals more often in spring–summer than in winter^3^.

Methods of bloodmeal analysis have evolved over time and have included immunological methods (precipitins, ELISA), DNA-based approaches (T-RFLP, PCR and Sanger sequencing, next generation sequencing) and protein characterization (liquid chromatography/mass spectrometry)^4–9^*.* Over the past two decades, a bloodmeal analysis method used widely across mosquitoes, triatomines, tsetse flies, sandflies, and ticks has been PCR-Sanger sequencing. The procedure typically involves the extraction of DNA from the excised, blooded abdomen of the insect. This DNA serves as a template to amplify a conserved vertebrate gene via PCR. Amplicons are directly sequenced by Sanger sequencing, and sequences are compared to known sequences using searches such as the Basic Local Alignment Search Tool [BLAST] search with National Center for Biotechnology Information [NCBI] GenBank^10^ to detect residual traces of vertebrate host DNA^5^. While this approach allows for species-specific host identification to the level of resolution afforded by the GenBank sequence database^10^, it has posed particular challenges in bloodmeal analysis of vectors with multiple bloodmeals and those that molt through several stages. For example, determination of bloodmeal sources of hard ticks is difficult due to degradation of vertebrate DNA while molting from one life stage to the next^11,12^. Bloodmeal analysis methods which detect only a single host may lead to a biased characterization of the host community. The widely used approach of PCR followed by direct Sanger sequencing does not efficiently detect multiple bloodmeal hosts or any bloodmeal other than the most abundant source at time of sampling. The rapid evolution of next generation sequencing makes it an attractive option for detecting multiple hosts fed upon by an individual vector.

Recently, amplicon deep sequencing has been used as a refinement to PCR-Sanger sequencing methods for identification of bloodmeal hosts^7,13–15^. In this approach, a conserved locus (frequently mitochondrial) that has nucleotide variation among taxa is amplified; amplicons are barcoded and sequenced on next generation sequencing platforms. This approach may yield hundreds of thousands of sequence reads per individual vector, as opposed to the single sequence trace generated using the Sanger approach. The use of amplicon deep sequencing in arthropod vector bloodmeal analysis has afforded the detection of: up to four vertebrate hosts in some *Rhodnius* vectors^14^, different individual humans in *Anopheles* mosquitoes^7^, non-human feeding in *Aedes aegypti* and *Culex quinquefasciatus*^16^, and simultaneous identification of host, ectoparasite, and pathogen DNA in ticks and fleas^17^.

Triatomine insects are obligate blood feeding arthropods that feed broadly on mammals (including humans), birds, reptiles, amphibians, and even invertebrates^4,18–22^. Triatomines are distributed throughout the Americas^23^, where they are vectors of *Trypanosoma cruzi*, the protozoan parasite responsible for Chagas disease. Over 5.7 million people across the Americas are estimated to suffer from Chagas disease^24^. In the southern United States, where eleven species triatomines are endemic, Chagas disease has been diagnosed in locally-infected humans^25–27^ and is recognized as a cause of cardiomyopathy and death in dogs^28–30^. Raccoons (*Procyon lotor* Linnaeus), woodrats (*Neotoma* spp.), opossums (*Didelphis virginiana* Kerr), and many other wildlife species have been recognized as reservoir hosts across the southern US^31^. Not only does bloodmeal source affect likelihood of an insect becoming infected with *T. cruzi*, but blood source has also been shown to affect vector life cycle duration and fecundity^32^ as well as proclivity to feed and molt^33^.

The objective of this study was to explore methods of characterizing the vertebrate host community of triatomines collected across Texas, a state with a high diversity of triatomine species and documented cases of locally-acquired human and canine infection^27,34–36^. We conducted a study of two molecular bloodmeal analysis approaches (direct Sanger sequencing and amplicon deep sequencing) in order to evaluate the application of these two sequencing approaches to the bloodmeal analysis of triatomine vectors of Chagas disease.

## Materials and methods

### Specimen collection

From June 2013 to December 2015, we acquired 115 triatomine specimens via two methods: 1) 45 specimens collected by community members across Texas, northern Mexico (USDA Import Permit 123470), and Florida and submitted to our community science program^37^, and 2) 70 specimens collected by co-authors and their teams using standard entomological trapping techniques, including lights, carbon dioxide, and active searching around houses, kennels and wood rat nests^37^. Of these 115 total samples, 60 triatomines had been previously dissected and tested for *T. cruzi* (see^38–40^ for details). We dissected 55 additional samples for this study (3 from Mexico, 39 from a non-human primate facility, and 13 from a zoological park—intending to test the breadth and resolution of species detection, including distinguishing between *Homo sapiens* and other primate species). In this study, our main goal was to assess sequencing methods; we therefore selected triatomine samples from areas with diverse host communities. This included specimens collected from a zoological park, as well as samples collected from non-human primate facilities. *T. cruzi* infection was detected by amplification of a 166 bp region of repetitive nuclear satellite DNA using a TaqMan qPCR reaction with Cruzi 1/Cruzi 2 primers and Cruzi 3 probe^41,42^ as previously described and applied to field-caught triatomines in Texas^39^.

### Sample preparation and *T. cruzi* molecular typing

Triatomines were stored at 4 °C until they were able to be identified^23^ and dissected as previously described, including submerging specimens in a 50% bleach solution prior to dissection to mitigate risk of human DNA contamination^43^. Based on visual examination of the dissected gut, evidence of a recent bloodmeal was scored (1 = no blood, desiccated guts; 2 = no blood, guts visible; 3 = traces of blood in gut; 4 = blood present, but either not much or not fresh [dried]; 5 = large amount of fresh blood)^38,43^; for the purposes of this study, these were further classified as ‘starved’ (scores of 1–3) or fed (scores of 4 or 5). After dissection, specimen guts were stored at − 20 °C and/or − 80 °C until extraction. DNA from hindgut tissue was extracted using the Omega E.Z.N.A Tissue DNA kit (Omega Bio-Tek, Norcross, GA). Samples were subjected to multiple PCRs for detection and strain-typing of *T. cruzi* DNA, as previously described^39^.

### PCR amplification of host Cytochrome b region

Conserved vertebrate DNA loci of 215 and 145 bp long from the mitochondrial 12S rRNA gene or 140 bp from the mammalian mitochondrial 16S rRNA have been used to identify vertebrate hosts from bloodmeals^7,13–15^. Similar to these methods, extracted hindgut DNA was subjected to PCR amplification of a 228 bp fragment of the vertebrate Cytochrome b gene^44,45^. The primers were originally designed to amplify reptilian hosts but have been reported to amplify a wide range of vertebrate hosts^45,46^. Reactions included 3 μL template DNA, primers at final concentrations of 0.66 μM each, and FailSafe PCR Enzyme Mix with PreMix E (Epicentre, Madison, WI) in a final volume of 50 μL. Primers used were ‘herp1’ 5'-GCH GAY ACH WVH HYH GCH TTY TCH TC-3' and ‘herp2’ 5'-CCC CTC AGA ATG ATA TTT GTC CTC A-3' and reactions were run with previously described cycling conditions for 55 cycles^44^. DNA-negative water controls and positive controls of DNA extracted from cynomolgus macaque (*Macaca fascicularis*) were included in each PCR batch. PCR amplicons were visualized on a 1.5% agarose gel stained with ethidium bromide.

Samples showing a band of ~ 228 bp, including some that also had an additional band of ~ 450 bp, were purified using ExoSAP-IT (Affymetrix, Santa Clara, CA). Triatomine dissection, DNA extraction, PCR, and post-PCR manipulations took place in separate dedicated areas in the laboratory in order to reduce risk of cross-contamination among samples,; in addition, three samples (PS334, PS675, PS1306) that revealed a human bloodmeal were processed further. A primer set specific to mammals (‘mammal a’ primer set) was used (5′-CGA AGC TTG ATA TGA AAA ACC ATC GTT G-3′ and 5′-TGT AGT TRT CWG GGT CHC CTA-3′)^46^. PCR reactions included 1.5 µl of DNA template with FailSafe PCR Enzyme Mix with PreMix E (Epicentre, Madison, WI). Denaturation was done at 95 °C for 5 minutes. Amplification cycles were done with denaturation at 95 °C for 30 seconds, annealing at 60 °C for 50 seconds and extension at 72 °C for 40 seconds for 36 cycles.

### Sanger sequencing of Cytochrome b amplicon

An aliquot of 5 μl of purified amplicon was sequenced with the ‘herp1’ primer using Sanger sequencing on a 3730xl DNA Analyzer (ThermoFisher Scientific, Waltham, MA) at Eton Bioscience Inc. (San Diego, CA). The remaining purified amplicon was stored at − 20 °C until further use. Sequence chromatograms were visually inspected for quality using 4Peaks (version 1.7.1) (Mekentosj, Amsterdam, http://www.nucleobytes.com/4peaks/). Sequences were compared to existing sequences in GenBank (https://www.ncbi.nlm.nih.gov/genbank/)^10^ using BLAST^47^ with default parameters to search in the ‘nucleotide collection (nr/nt)’ database—which includes GenBank, EMBL, DDBJ, PDB, RefSeq and excludes EST, STS, GSS, WGS, TSA—for ‘highly similar sequences (megablast)’ (https://blast.ncbi.nlm.nih.gov/Blast.cgi). In contrast to a previous study which set a threshold for accepting and reporting a result at ≥ 95% identities and E-value ≤ 0^20^, we set a more liberal threshold to include samples with ≥ 90% identities as a preliminary identification and criterion for moving to amplicon deep sequencing, in order to further evaluate lower identity matches by amplicon deep sequencing. We also considered biological feasibility, defined as the possibility of host presence at the collection site of the triatomine vector, given the current understanding of host species distributions and occurences (Texas Parks and Wildlife https://tpwd.texas.gov/ and Austin Zoo https://austinzoo.org/).

### Amplicon deep sequencing and bioinformatics analyses

For all samples yielding ≥ 90% identity BLAST matches based on direct Sanger sequencing, the remaining volume of purified PCR product (~ 30 µL) for each sample was subjected to amplicon deep sequencing at Texas A&M AgriLife Research Genomics and Bioinformatics Service (College Station, TX). PCR products from the herp1/herp2 reactions were made into Illumina-compatible sequencing libraries by addition of adapters and indexes in two sequential PCR reactions.

In the first reaction, Illumina-based sequence read primers were added to the initial herp-1 PCR products using a common reverse primer (Herp_2R, 5'-GTG ACT GGA GTT CAG ACG TGT GCT CTT CCG ATC TCC CCT CAG AAT GAT ATT TGT CCT CA-3') and one of four padded forward primers designed to add diversity for increased data yield and quality. The forward primers used (with bases added for diversity underlined) were Herp_1’A, 5'-ACA CTC TTT CCC TAC ACG ACG CTC TTC CGA TCT GCH GAY ACH WVH HYH GCH TTY TCH TC-3'; Herp_1’B, 5'-ACA CTC TTT CCC TAC ACG ACG CTC TTC CGA TCT HGC HGA YAC HWV HHY HGC HTT YTC HTC-3'; Herp_1’C, 5'-ACA CTC TTT CCC TAC ACG ACG CTC TTC CGA TCT HWG CHG AYA CHW VHH YHG CHT TYT CHT C-3'; and Herp_1’D, 5'-ACA CTC TTT CCC TAC ACG ACG CTC TTC CGA TCT HWW GCH GAY ACH WVH HYH GCH TTY TCH TC-3'. Each 25 µl reaction contained 1 ng herp1 PCR product, 0.5 µM each forward and reverse primer, 200 µM dNTPS, and 0.02 U/µl Phusion Taq DNA polymerase (New England Biolabs, Ipswich, MA, USA) in 1X Phusion reaction buffer. Samples were initially denatured at 98 °C for 30 s, then cycled eight times at 98 °C for 10 s, 58 °C for 20 s, and 72 °C for 30 s. Samples were held at 10 °C following a final elongation for 5 min at 72 °C, bead purified with 1X AMPure XP beads (Beckman Coulter Indianapolis, IN, USA), quantified with picogreen reagent (ThermoFisher Scientific Waltham, MA, USA), and checked for size on a fragment analyzer (Agilent, Santa Clara, CA, USA).

The second PCR added combinatorial dual indexes with the following sequences (where X represents barcode bases): P5_Index_Primer, 5'-AAT GAT ACG GCG ACC ACC GAG ATC TAC ACX XXX XXX XAC ACT CTT TCC CTA CAC GAC GCT CTT CCG ATC T-3' and P7_Index_Primer, 5'-CAA GCA GAA GAC GGC ATA CGA GAT XXX XXX GTC TCG TGG GCT CGG-3'. PCR reaction components and thermocycling conditions were similar to the first round, but unique combinations of a P5 and P7 index primer were used instead of Illumina-herp1 primers. PCR products were cleaned, quantified, and visualized as described above, and equimolar amounts of barcoded final PCR products were pooled and quantified using the Kappa library quantification qPCR kit. Pooled libraries were sequenced on a MiSeq (Illumina, San Diego, CA, USA).

Demultiplexed data were filtered and clustered to remove errors using SeekDeep bioinformatics pipeline (version 2.5.0)^48^. Sequences for each sample were filtered for minimal length (228 bp), 97% identity and quality (Phred score of above 25 across 75% of the read length). Clusters with reads less than 0.5% relative abundance were rejected. The rest of the output consensus sequences were subjected to taxonomy search using nucleotide BLAST^47^ using default parameters to search in the ‘nucleotide collection (nr/nt)’ database for ‘highly similar sequences (megablast)’; we considered accepted matches (here referred to as ‘usable clusters’) generated by amplicon deep sequencing to be those with > 99% identity and 100% query cover. As with the Sanger sequencing analyses, we considered the biological feasibility of the results.

## Results

### Triatomine collection and *T. cruzi* infection in triatomines

The 115 triatomines in this study were collected between June 2013 and October 2015 and included seven species of *Triatoma*: *T. gerstaeckeri* (Stål) (84; 73.0%), *T. sanguisuga* (Leconte) (16; 13.9%), *T. lecticularia* (Stål) (6; 5.2%), *T. indictiva* Neiva (4; 3.5%), *T. mexicana* (Herrich-Schaeffer) (1; 0.9%), *T. neotomae* Neiva (1; 0.9%), 1 unidentified *Triatoma* adult (0.9%), and 2 *Triatoma* nymphs (unknown species; 1.7%). These samples were collected primarily across 22 counties in Texas, with the exception of one sample from Florida (Lake County) and three samples from Mexico (states of Hidalgo and Tamaulipas). Habitats from which insects were collected included the grounds of a zoo, a nonhuman primate research facility, in and around private residences, dog kennels, chicken coops, farms, and tents. Samples represented different bloodmeal conditions identified at the time of dissection: 44 samples (38.3%) had a bloodmeal score of 4 or 5 and were considered ‘fed’. A subset of 71 triatomines were reported to be alive at time of collection, while 44 were dead prior to collection. Of the 115 triatomines, 64 (55.7%) were infected with *T. cruzi*. *T. cruzi* strains in infected triatomines were: 35 TcI (54.7%), 23 TcIV (35.9%), and 6 (9.4%) TcI/TcIV mixed infections.

### PCR amplification of Cytochrome b region

We attempted to amplify the a 228 bp region of the Cytochrome b gene from hindgut DNA extracts of 115 triatomines. Overall, 56 generated bands—38 samples (11 ‘starved’ triatomines and 27 ‘fed’ triatomines) generated single bands of the target amplicon size (228 bp); an additional 18 samples (16 ‘starved’ triatomines and 2 ‘fed’ triatomines) generated a band of approximately 450 bp along with the expected band of the 228 bp target amplicon size.

### Sanger Sequencing

All 38 samples associated with a single band were submitted for Sanger sequencing, 36 of which produced a sequence resulting in identification (≥ 90% identities and expect (E) values of ≤ 0) in NCBI BLAST (Table 1). We attempted Sanger sequencing of 15 of the 18 samples that had generated two bands; only 2 samples of these samples were successfully sequenced via Sanger. Attempts to investigate what the ~ 450 bp product might be by cutting only the ~ 450 bp products from the gels with two bands and generating sequences from the larger fragment were unsuccessful.

**Table 1:** Triatomine bloodmeal scores, number of samples with hosts identified, and detection of multiple bloodmeal hosts via next generation sequencing.

	n	~ 228 bp band on PCR	Sanger sequencing produced appropriately sized fragment that resulted in a BLAST match	Single host detected by NGS	Multiple hosts detected by NGS	Identifiable host results	95% confidence interval of identifiable host results
**Bloodmeal status**							
Starved	71	27	10	7	3	14.1%	7.8–24.0%
Fed	44	29	26	24	2	59.1%	44.4–72.3%
Total	115	56	36	31	5	31.3%	23.6–40.3%
**Collection status**							
Alive	71	35	23	20	3	32.4%	22.7–43.9%
Dead	44	21	13	11	2	29.5%	18.2–44.2%
Total	115	56	36	31	5	31.3%	23.6–40.3%

During dissection, triatomines were assigned bloodmeal scores of 1 (no blood, desiccated guts), 2 (no blood, guts visible), 3 (traces of blood in gut), 4 (blood present, but either not much or not fresh [dried]), or 5 (large amount of fresh blood); these were then classified as ‘starved’ (scores of 1–3) or fed (scores of 4 or 5). The herp1/herp1 primer set was used to amplify a 228 bp fragment of DNA (some samples also produced a second band of ~ 450 bp). Sanger sequencing and BLAST matching were used to determine bloodmeal hosts. Triatomines of ‘fed’ statuses more frequently had a bloodmeal host characterized via Sanger sequencing that triatomines of ‘starved’ statuses. In addition to Sanger sequencing, amplicons were subjected to next generation sequencing methods. Five triatomines had multiple hosts detected via next generation sequencing methods.

Of the 44 samples from triatomines that appeared ‘fed’ at time of dissection, 26 (59.1%; 95% CI 44.4–72.3%) yielded identifiable host results via PCR and Sanger sequencing (Table 1). In contrast, only 10 of the 71 triatomines that appeared ‘starved’ yielded identifiable host results (14.1%; 95% CI 7.8–24.0%) (df = 1, Pearson χ^2^ = 25.59, *p* < 0.001).

Of the 71 samples from triatomines that had reportedly been collected alive, 23 (32.4%; 95% CI 22.7–43.9%) yielded identifiable host results via PCR and Sanger sequencing (Table 1). In comparison, 13 of the 44 triatomines that had been collected when already dead yielded identifiable host results (29.5%; 95% CI 18.2–4.2%) (df = 1, Pearson χ^2^ = 0.1, *p* = 0.75).

In the 36 samples that generated a preliminary identification by Sanger sequencing, fifteen vertebrate species were identified, including 12 mammalian species, one bird, one reptile, and one amphibian (Table 2). In order of frequency detected: *Canis lupus familiaris* (domestic dog - 11), *H. sapiens* (human - 5), *Gallus gallus* (chicken - 3), *Ovis aries* (sheep - 3), *Incilius nebulifer* (Gulf Coast toad - 2), *Panthera tigris* (tiger - 2), *Rattus rattus* (black/roof rat - 2), *Bos taurus* (cattle - 1), *Capra hircus* (goat - 1), *Chelonoidis carbonaria* (red-footed tortoise - 1), *Colobus* spp (colobus monkey - 1), *Felis catus* (cat - 1), *Odocoileus virginianus* (white-tailed deer - 1), *Sciurus carolinensis* (eastern gray squirrel - 1), and *Sciurus niger* (fox squirrel - 1).

**Table 2:** Triatomine sample information for specimens with bloodmeal host result determined by direct Sanger and NGS methods.

	Sample ID	*Triatoma* species	Collection Area	*T. cruzi* status	*T. cruzi* DTU	Bloodmeal score	Sanger sequencing			Next generation sequencing				
							Sanger sequencing result	E-value	% Identity	Next generation sequencing result	Taxon assigned reads	Relative abundance (%)	E-value	% Identity
Triatomines with only one bloodmeal host revealed	AZ-011	*T. gerstaeckeri*	Zoo—redfooted tortoise area	Positive	TcI/TcIV	4	*Chelonoidis carbonaria*	3.0E−93	98	*Chelonoidis carbonaria*	164,352	100	9.0E−104	100
	AZ-036	*T. gerstaeckeri*	Zoo—leopard area	Positive	TcIV	2	*Rattus rattus*	2.0E−94	99	*Rattus rattus*	309,811	100	9.0E−104	100
	AZ-043	*T. sanguisuga*	Zoo—wolf hybrid area	Negative	–	1	*Gallus gallus*	2.0E−94	98	*Gallus gallus*	226,082	100	9.0E−104	100
	AZ-082	*T. sanguisuga*	Zoo—lion area	Negative	–	4	*Rattus rattus*	5.0E−96	99	*Rattus rattus*	470,378	100	9.0E−104	100
	AZ-084	*T. gerstaeckeri*	Zoo—unknown exact area	Negative	–	5	*Colobus spp*	7.0E−95	98	*Colobus guereza*	453,712	100	9.0E−104	100
	AZ-118	*T. indictiva*	Zoo—tiger area	Negative	–	3	*Panthera tigris*	2.0E−79	96	*Panthera tigris*	91,503	100	9.0E−104	100
	EL016	*T. gerstaeckeri*	Near house with dog	Positive	TcI	3	*Canis lupus familiaris*	8.0E−69	93	*Canis lupus familiaris*	57,653	100	9.0E−104	100
	PS195	*T. gerstaeckeri*	Dog kennel	Positive	TcI	4	*Canis lupus familiaris*	9.0E−94	98	*Canis lupus familiaris*	511,553	100	9.0E−104	100
	PS253	*T. gerstaeckeri*	Dog kennel	Positive	TcI	5	*Canis lupus familiaris*	2.0E−96	97	*Canis lupus familiaris*	453,871	100	9.0E−104	100
	PS334	*T. sanguisuga*	On person outdoors	Positive	TcIV	5	*Homo sapiens*	1.0E−97	99	*Homo sapiens*	511,705	100	9.0E−104	100
	PS403	*T. sanguisuga*	In house with dog	Positive	TcIV	4	*Canis lupus familiaris*	6.0E−95	100	*Canis lupus familiaris*	471,446	100	9.0E−104	100
	PS449	*T. gerstaeckeri*	In house—in bed	Positive	TcI	5	*Homo sapiens*	3.0E−88	98	*Homo sapiens*	269,282	100	9.0E−104	100
	PS498	*T. gerstaeckeri*	Farm	Positive	TcI	4	*Incilius nebulifer*	3.0E−98	99	*Incilius nebulifer*	525,182	100	9.0E−104	100
	PS501	*T. gerstaeckeri*	On house on farm with sheep	Positive	TcI	2	*Ovis aries*	5.0E−86	97	*Ovis aries*	260,955	100	9.0E−104	100
	PS503	*T. gerstaeckeri*	Farm with sheep	Positive	TcI	4	*Ovis aries*	2.0E−85	97	*Ovis aries*	406,253	100	9.0E−104	100
	PS507	*T. gerstaeckeri*	Farm with goats	Positive	TcIV	4	*Capra hircus*	4.0E−87	98	*Capra hircus*	124,980	100	9.0E−104	100
	PS675	Unknown (nymph)	House	Negative	–	5	*Homo sapiens*	3.0E−93	99	*Homo sapiens*	475,763	100	9.0E−104	100
	PS888	*T. gerstaeckeri*	Farm with chickens	Negative	–	4	*Gallus gallus*	8.0E−89	98	*Gallus gallus*	536,574	100	9.0E−104	100
	PS889	*T. gerstaeckeri*	Farm with cats	Negative	–	3	*Felis catus*	1.0E−66	99	*Felis catus*	59,190	100	9.0E−104	100
	PS1035	*T. gerstaeckeri*	Unknown	Positive	TcIV	5	*Canis lupus familiaris*	1.0E−87	99	*Canis lupus familiaris*	357,283	100	9.0E−104	100
	PS1102	*T. gerstaeckeri*	Outside of house with dog	Positive	TcIV	5	*Canis lupus familiaris*	3.0E−93	98	*Canis lupus familiaris*	357,533	100	9.0E−104	100
	PS1119	*T. lecticularia*	Chicken coop	Negative	–	4	*Gallus gallus*	1.0E−87	98	*Gallus gallus*	272,724	100	9.0E−104	100
	PS1122	*T. gerstaeckeri*	House with dog	Positive	TcIV	4	*Canis lupus familiaris*	4.0E−97	99	*Canis lupus familiaris*	357,749	100	9.0E−104	100
	PS1156	*T. gerstaeckeri*	Dog bed	Positive	TcI/TcIV	4	*Canis lupus familiaris*	2.0E−96	100	*Canis lupus familiaris*	257,656	100	9.0E−104	100
	PS1164	*T. gerstaeckeri*	Farm with cattle	Positive	TcI	5	*Bos taurus*	5.0E−86	98	*Bos taurus*	326,466	100	9.0E−104	100
	PS1256	*T. indictiva*	House with dog	Positive	TcI	5	*Canis lupus familiaris*	2.0E−95	99	*Canis lupus familiaris*	481,273	100	9.0E−104	100
	PS1257	*T. gerstaeckeri*	Near dog	Positive	TcI/TcIV	4	*Canis lupus familiaris*	5.0E−91	98	*Canis lupus familiaris*	443,378	100	9.0E−104	100
	PS1281	*T. gerstaeckeri*	In house with dog	Positive	TcI	5	*Canis lupus familiaris*	3.0E−93	98	*Canis lupus familiaris*	500,946	100	9.0E−104	100
	PS1301	*T. lecticularia*	House	Negative	–	5	*Homo sapiens*	1.0E−91	99	*Homo sapiens*	340,185	100	9.0E−104	100
	PS1306	*T. sanguisuga*	House	Negative	–	5	*Homo sapiens*	1.0E−96	98	*Homo sapiens*	402,405	100	9.0E−104	100
	SNBL005	*T. gerstaeckeri*	Non-human primate research center	Negative	–	2	*Odocoileus virginianus*	7.0E−55	96	*Odocoileus virginianus*	8,453	100	4.0E−102	99
Triatomines with more than one bloodmeal host revealed	AZ-040	*T. indictiva*	Zoo—tiger area	Negative	–	1	*Panthera tigris*	2.0E−69	94	*Panthera tigris*	47,708	96.3	9.0E−104	100
										*Homo sapiens*	1818	3.7	9.0E−104	100
	AZ-085	*T. sanguisuga*	Zoo—fox area	Negative	-	3	*Incilius nebulifer*	2.0E−48	92	*Incilius nebulifer*	1021	92.6	9.0E−104	100
										*Homo sapiens*	36	3.3	9.0E−104	100
										*Canis lupus familiaris*	24	2.2	9.0E−104	100
										*Sciurus carolinensis*	22	2.0	4.0E−102	99
	PS393	*T. sanguisuga*	On outside of house	Positive	TcIV	4	*Sciurus niger*	1.0E−76	97	*Sciurus niger*	15,622	90.6	9.0E−104	100
										*Homo sapiens*	1625	9.4	9.0E−104	100
	PS502*	*T. gerstaeckeri*	Farm with sheep	Positive	TcI	2	*Ovis aries*	2.0E−75	94	*Ovis aries*	67,481	94.4	9.0E−104	100
										*Bassariscus astutus*	4014	5.6	9.0E−104	100
	PS706	*T. sanguisuga*	On outside of house	Positive	TcIV	4	*Sciurus carolinensis*	8.0E−89	98	*Sciurus carolinensis*	247,455	99.3	4.0E−102	99
										*Procyon lotor*	1762	0.7	9.0E−104	100

*998 reads in this sample identified *Colobus guereza*. The plausibility of this host in this sample was not verifiable and therefore, these were set aside from consideration.

Ecological data and vertebrate bloodmeal host identifications based on direct Sanger sequencing versus amplicon deep sequencing for 36 triatomines collected across Texas and Florida.

Three samples (PS334, PS675, PS1306) revealed a potential human bloodmeal using the ‘herp’ primers. These were then subjected to PCR using the ‘mammal a’ primers, which also revealed human host results. (E-values of ≤ 0 and 99% identities in NCBI BLAST matches for all samples).

### Amplicon deep sequencing

Amplicon deep sequencing analysis was performed on 36 samples for which Sanger sequences were obtained (32 samples with ≥ 95% identities discovered by BLAST on Sanger sequencing products, and an additional 4 samples with < 95% identities—ranging from 92 to 94%—in order to evaluate lower identity matches using amplicon deep sequencing). We obtained 22,155,506 reads (Supplemental Table 1) from the MiSeq run, of which 18,236,359 reads matched Illumina adapters and were used in further filtering. When filtered by size and quality scores, 11,023,747 sequences were available for clustering; of these, 10,874,884 reads matched a vertebrate host species in GenBank. In total, 49.8% of the raw reads and 60.4% of the adapter matched reads were usable, with an average of 598,764 raw and 308,080 usable reads per sample.

Seventeen vertebrate host species were identified by amplicon deep sequencing (Table 2). In order of frequency detected: *C. lupus familiaris* (dog - 12), *H. sapiens* (human - 8), *G. gallus* (chicken - 3), *O. aries* (sheep - 3), *I. nebulifer* (Gulf Coast toad - 2), *P. tigris* (tiger - 2), *R. rattus* (black/roof rat - 2), *B. astutus* (ringtail - 1), *B. taurus* (cow - 1), *C. hircus* (goat - 1), *C. carbonaria* (red-footed tortoise - 1), *Colobus guereza* (guereza - 1), *F. catus* (cat - 1), *O. virginianus* (white-tailed deer - 1), *P. lotor* (raccoon - 1), *S. carolinensis* (eastern gray squirrel - 1), and *S. niger* (fox squirrel - 1). Of the 12 samples that indicated dog as a blood source, 11 were infected with *T. cruzi*—5 with TcI, 4 with TcIV, and 2 with TcI/TcIV mixed infections. Of the 8 samples that indicated human as a blood source, 3 (38%) were infected with *T. cruzi*—1 with TcI and 2 with TcIV (Table 2).

In the BLAST matches, the output consensus sequences matched perfectly (100% identity) across the entire target in all but three instances (99% for *S. carolinensis* [Eastern gray squirrel; AZ-085 and PS706] and *O. virginianus* [white-tailed deer; SNBL005]; Table 2). Samples rejected on the basis of a poor match showed 53% or lower coverage (data not shown). A finding from one sample was rejected on the basis of a lack of biological feasibility (PS502) in which 998 (1.38%) of 72,493 reads matched with 100% identity to *C. guereza* (eastern black and white colobus); the other two hosts identified for this sample were *O. aries* (domestic sheep; 93.09% of reads and 100% identity match) and *Bassaricus astutus* (ringtail; 5.54% of reads and 100% identity match). This triatomine was found on a rural ranch in Gillespie County, Texas, where the likelihood of finding the *Colobus* host was remote. The reads matching the *Colobus*, were, therefore curated out of further analysis on the basis of biological implausibility.

### Comparison of Sanger sequencing and amplicon deep sequencing results

**Figure 1:**
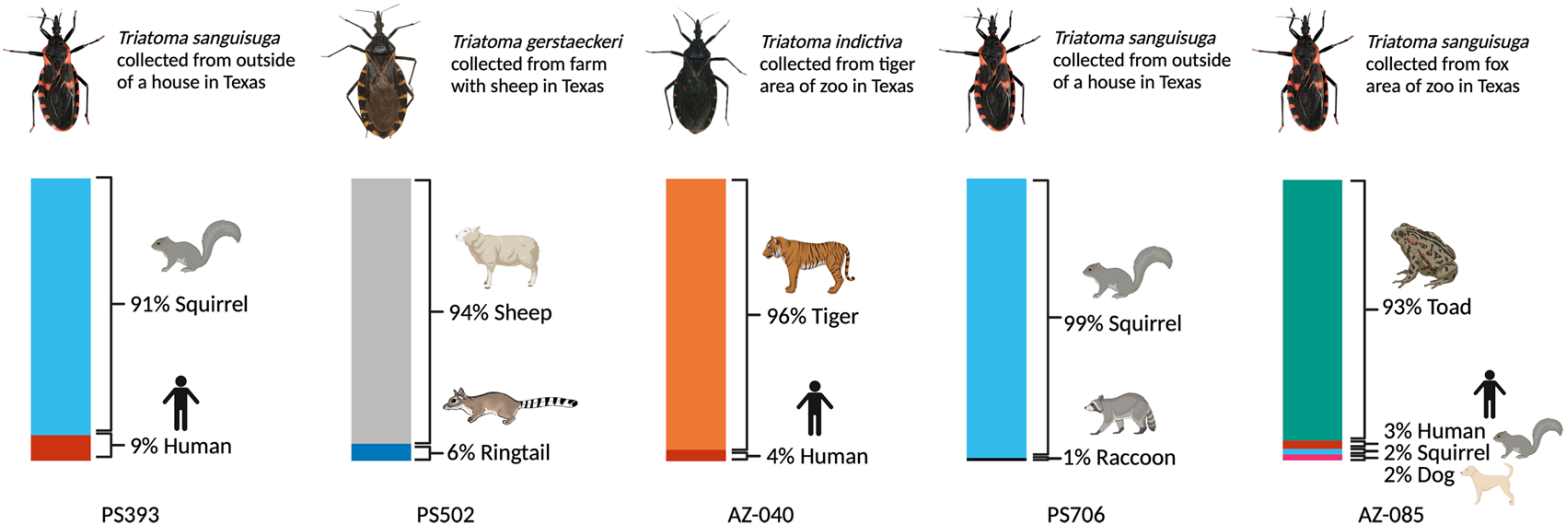
Amplicon deep sequencing revealed multiple bloodmeal sources in several triatomines. DNA extracted from triatomine hind guts was subjected to amplicon deep sequencing. Of the 36 samples generating amplicon deep sequencing results, 5 samples generated results revealing more than one bloodmeal source. One of the five samples had four bloodmeal sources identified. Ringtail image created by Dr. Tara Roth. Figure created with https://biorender.com/.

While some differences were observed in the frequency of occurrence of hosts between Sanger sequencing and amplicon deep sequencing, the most abundant host identified by both methods was domestic dog (30.6% by Sanger sequencing and 33.3% by amplicon deep sequencing, Table 2). The next most frequent host was human (13.9% by Sanger sequencing and 22.2% by amplicon deep sequencing). Amplicon deep sequencing not only replicated the host dataset as determined by Sanger sequencing, but also afforded the detection of additional hosts within a subset of triatomines (Table 2). Five triatomines (13.9% of the 36 vectors for which host was determined) had evidence of multiple hosts; four showed evidence of blood from two species and one showed evidence of blood from four species (Fig. 1). In all samples with multiple hosts detected, a dominant amplicon represented > 90% of reads (Table 2), which was also the taxon identified by Sanger sequencing. Species represented in triatomines with evidence of multiple bloodmeals included domestic (human, domestic dog, sheep) and wildlife species (Gulf coast toad, tiger [from a zoo], Eastern gray squirrel*,* fox squirrel*,* ringtail, raccoon).

## Discussion

We used two techniques—direct Sanger sequencing and amplicon deep sequencing—to identify hosts fed upon by triatomines collected primarily from Texas. Our findings of diverse wildlife species, domestic dog, and human bloodmeal sources corroborate previous findings of opportunistic feeding habits of some species of triatomines found in the US. Amplicon deep sequencing was able to identify up to 4 different blood sources within one triatomine, compared to Sanger sequencing alone only identifying a single, and likely most recent, source within each triatomine.

Triatomines are long-lived insects that feed multiple times in each of five nymphal instars and throughout the adult life stage^23,49^. For triatomine bloodmeal analysis, direct Sanger sequencing of a single PCR product—a method which typically reveals only the most abundant host taxa—may fail to provide the information necessary to learn about transmission networks. PCR followed by cloning and sequencing has offered more success detecting multiple bloodmeals^18,50,51^. Amplicon deep sequencing fills the need for a powerful method that detects not only the most recent/abundant host DNA, but also older and partially degraded bloodmeals as well. This method is becoming increasingly used in the detection of bloodmeals from triatomines yielding multiple host information from individual bugs allowing comparison of triatomines from different habitats, understanding their behaviors and controlling the spread of Chagas disease^13–15,52^.

Despite many advantages, the limits of amplification-based methods include that a single primer pair may not capture and amplify from all existing taxa with equal efficiency. For this reason, the ratios of the reads may not represent the ratio of the host abundance within the bloodmeal and conclusions to this effect are to be drawn with caution.

The primers used in this study amplify a small region (228 bp) which helps increase likelihood of detecting older, degraded bloodmeals. However, even with this small target, only 56 of 115 (48.7%) of samples produced an amplicon of the expected 228 bp size, and only 36 of those (31.3% overall) resulted in a sequenced host identification. As noted previously^20^, samples with noticeable blood were more likely to result in host identification (59.1%), and those samples that were starved had a much lower success rate for both PCR product detection and host identification (14.1%) (Table 1). Researchers aiming to maximize their success rate may be interested in focusing on processing samples from triatomines with evidence of bloodmeals at time of dissection. Interestingly, of the five samples that generated multiple host results, three had been classified as ‘starved’ at time of dissection. More research is needed to understand whether the ability to detect multiple hosts differs between samples with and without evidence of bloodmeals at time of dissection—processing ‘starved’ samples could be valuable to detect degraded bloodmeals from multiple hosts. Although we regularly use primer sets for other vertebrate genes in our vector bloodmeal analysis work, our prior (unpublished) data have shown greatest success with the herp primer set which was used in the current study. Multiple PCRs and longer fragments are likely to increase the certainty of detection of host and also potentially allow for detection of diverse hosts (although at increased processing/analysis costs). However, a shorter fragment is more likely to amplify DNA from degraded blood in starved, field caught triatomines. In addition, a long fragment (> ~ 600 bp) would require paired end sequencing, and the number of reads that can be dedicated towards one sample would be reduced when conducting next generation sequencing. Future studies might explore success using other primer sets.

Although the triatomines in this study had some variation in whether they had been collected alive or dead, we did not find a difference in success rate for PCR product detection and host identification for samples collected alive versus those collected dead (Table 1), which has practical implications for community science programs where keeping the vector alive is not advisable and where dead vectors may have been stored and mailed in variable conditions. We showed successful host identification using both Sanger and amplicon deep sequencing from insects that were collected and stored by members of the public under variable conditions, including insects that were found dead and not dissected until ~ 210–400 days after collection (AZ-36, AZ-40, AZ-43, and PS498). The methods used here, although they did not generate results for many samples, were able to generate results for some samples that had been stored in non-optimal conditions prior to processing.

An additional consideration when choosing a bloodmeal analysis method is cost per sample. In general, next generation sequencing methods require more expensive instruments and consumables, and bioinformatics training to analyze the resulting data, making them more expensive than Sanger sequencing. As technology becomes more accessible and affordable, amplicon deep sequencing may be useful for addressing research questions focusing on host-vector interactions in systems where vectors feed on multiple hosts over time.

Triatomines require multiple bloodmeals to molt and reach adulthood. Evidence of multiple host taxa within individual triatomines—including up to four different bloodmeal sources in one triatomine—have been detected using antisera^3,6^, ELISAs^53,54^, species-specific primer sets^55,56^, and PCR and cloning^18,50^. Detection of multiple bloodmeal sources using PCR and direct sequencing is rare, although has occurred in at least one triatomine collected in Texas^20^. Next generation sequencing has recently been shown to be a sensitive method for detecting an average of 4.9 bloodmeal sources per triatomine^15^. Much remains to be explored regarding how long evidence of a bloodmeal source can be found in a triatomine, and whether detection of multiple bloodmeal sources is indicative of meals over several stages or partial/incomplete feeding attempts during one life stage of a specimen. The five individuals generating multiple host identifications (4.3%) in this study had a variety of fed and starved statuses. In order to better understand how host feeding patterns inferred from these bloodmeal analysis methods reflect true timing and sequence of triatomine feeding in nature, we suggest experimental studies investigating detection rates of hosts after controlled feeding of different sources of blood to triatomines in laboratory colonies.

Our findings add to the evidence of some species of triatomines found in the US as opportunistic and indiscriminate feeders. Although some triatomines may be sit-and-wait nest specialists with strong host associations and others have more active foraging behaviors and are host generalists^57^, much remains to be learned about the behaviors of many triatomine species found in the southern US. In the southern US, the species in our study are typically considered sylvatic and are predominantly maintained by wildlife species. A recent quantitative synthesis from 14 published bloodmeal analysis studies of triatomines found in the US showed at least 44 host taxa/host groups represented among 449 insects, emphasizing their opportunistic and indiscriminate feeding behaviors^58^.

Triatomines used in this study were mainly collected by community scientists in/near homes, and those samples predictably revealed bloodmeals including human and animals associated with domestic and peridomestic environments (dog, cattle, cat, chicken, sheep, and goat). The most frequently identified bloodmeal source was dog (33.3% of triatomines with any host detected contained dog blood); 11 of the 12 triatomines with dog blood revealed had only dog blood. The second most frequently identified bloodmeal source was human; this included 5 triatomines with only human blood and 3 triatomines with human blood and another species. The 3 triatomines with a small percentage of reads (3.4–9.4%) indicating human results, as well as the biologically implausible finding of *Colobus* DNA in a sample found in rural Texas (PS 502), raise concern for potential contamination of samples during processing. The possibility of contamination was much reduced by strict procedures to minimize and monitor for contamination, including separate pre- and post-PCR processing areas, bleaching of triatomines prior to dissection to remove exogenous DNA, and inclusion of negative controls in PCRs. These findings of small amounts of human DNA in samples may reflect a less abundant or less recent feeding on humans. Using highly-sensitive techniques, such as the methods used here, increases the likelihood of detecting contamination in samples; all results should be considered with respect to the actual probability that the bloodmeal hosts revealed by the methods are biologically feasible.

The most frequently identified bloodmeal source was dogs, which are recognized reservoirs of *T. cruzi* in Texas^29,36^. Of the 12 samples that indicated dog as a blood source, 11 were infected with *T. cruzi*—5 with TcI, 4 with TcIV, and 2 with TcI/TcIV mixed infections. Of the 8 samples that indicated human as a blood source, 3 (33%) were infected with *T. cruzi*—1 with TcI and 2 with TcIV—representing potential infection risk if the person had been exposed to the triatomine feces (alternately, it could indicate the triatomine had fed on an infected human, but this likelihood is low in the US). Known wildlife reservoirs of *T. cruzi*^31^—squirrels, ringtails, raccoons—were determined to be bloodmeal sources in this sample set. Triatomines in zoos and *T. cruzi* infection in zoo animals have been previously documented^59,60^. In the current study, several triatomines from a zoo harbored a variety of mammalian hosts—tiger, colobus monkey, roof rat, humans and dogs—capable of being infected by *T. cruzi*, as well as non-mammalian hosts—Gulf coast toad, red-footed tortoise, chicken—refractory to *T. cruzi* infection (although see^61,62^). Two triatomines that had fed on red-footed tortoise and Gulf coast toad were infected with *T. cruzi*, indicating these triatomines likely had meals from reservoirs prior to feeding on these potentially refractory species. Feeding studies of laboratory-reared bugs with known and changing bloodmeals over the triatomine lifecycle, and across the long duration of the adult stage, will be key to interpretation of future findings^20^. Advances in bloodmeal determination are needed to further explore the wild sources of *T. cruzi* infection in triatomines prior to their dispersal to human houses and their surroundings where they pose public and veterinary health risk.

Amplicon deep sequencing has potential as a powerful technique for elucidating multi-host feeding patterns of triatomines, and additional research focused on factors—such as engorgement status—predicting host detection success will aid in optimized sample selection and processing. As additional knowledge of blood feeding patterns is generated, a more intricate understanding of vector ecology and bloodmeal sources can be coupled with *T. cruzi* reservoir infection data, which may be useful for designing disease risk reduction interventions.

## Supplementary Information

Supplementary Information.

## Data Availability

Data from next generation sequencing have been deposited into the SRA database (NCBI) under the accession numbers SRR19358211 to SRR19358246.
