# Supplementary material for: Characterization of triatomine bloodmeal sources using direct Sanger sequencing and amplicon deep sequencing methods
Source: Sci Rep. 2022 Jun 17;12:10234. doi: 10.1038/s41598-022-14208-8 (PMC9205944; doi:10.1038/s41598-022-14208-8)
Supplement: Supplementary file 1 — Supplementary Information. [file 41598_2022_14208_MOESM1_ESM.docx]

**SUPPLEMENTAL TABLE 1** Next generation sequencing reads summary. Run was analyzed using the SeekDeep bioinformatics pipeline. Parameters were for length at 228 bp, and quality score above 25 across 75% of the read. Reads were clustered based on 97% identity eliminating clusters in lowest 0.5% by relative abundance. Taxon identifiable reads filtered out biologically unfeasible reads and were used in the reporting of vertebrate hosts.

|  | Total Reads | Highest Reads Per Sample | Lowest Reads Per Sample | Average | Standard Deviation |
| --- | --- | --- | --- | --- | --- |
| Raw reads | 21,555,506 | 917,842 | 410,580 | 598,764 | 106,374 |
| Illumina adapter identified reads | 18,236,359 | 847,112 | 344,718 | 506,565 | 102,386 |
| Failed quality filtering | 7,168,894 | 716,490 | 11,521 | 199,135 | 213,737 |
| Failed primer | 43,718 | 1,973 | 608 | 1,214 | 314.32 |
| Reads used in clustering | 11,023,747 | 536,853 | 1,454 | 306,215 | 166,882 |
| Reads identifying host | 10,874,884 | 536,574 | 1,103 | 302,080 | 170,956 |
